# Integrative analysis of small RNA and degradome sequencing reveals the role of miRNAs in monoterpene biosynthesis in linalool-type *Cinnamomum camphora*

**DOI:** 10.1186/s12870-025-07588-2

**Published:** 2025-11-11

**Authors:** Hao Rong, Qiaoli Liu, Zhaoxiang Wu, Huihu Li, Pengzhenni Chen, Yongjie Zheng, Yongda Zhong, Caihui Chen

**Affiliations:** 1 Jiangxi Provincial Key Laboratory of Improved Variety Breeding and Efficient Utilization of Native Tree Species, Institute of Resources and Environment, Jiangxi Academy of Sciences, No. 7777, Changdong Road, Gaoxin District, Nanchang, 330096 China; 2 Jiangsu Academy of Agricultural Sciences Wuxi Branch, Wuxi, 214171 China; 3 Camphor Engineering Technology Research Center for National Forestry and Grassland Administration, Jiangxi Academy of Forestry, Nanchang, Jiangxi 330032 China

**Keywords:** *Cinnamomum camphora*, MiRNA, Terpenoids, Linalool

## Abstract

**Background:**

*Cinnamomum camphora* is a valuable aromatic oil-producing species with significant economic and industrial importance. Linalool, a monoterpenoid compound and a major component of camphor leaf essential oil, is widely used in cosmetics, food, and pharmaceuticals. While microRNAs (miRNAs) are known to regulate terpenoid biosynthesis, their regulatory role in linalool biosynthesis remains largely unexplored.

**Results:**

In this study, we performed small RNA and degradome sequencing on three *C. camphora* samples (H_MAR, H_MAY, and L_MAY) exhibiting significant differences in linalool content. A total of 199 known and 200 novel miRNAs were identified. Among them, 170 differentially expressed miRNAs (DEMs; 83 downregulated and 87 upregulated) were detected between H_MAY and H_MAR, whereas 77 DEMs (45 downregulated and 32 upregulated) were found between H_MAY and L_MAY. Degradome analysis predicted 223 target genes for 52 known miRNAs and 86 targets for 39 novel miRNAs. Network analysis revealed that the miRNA-SPL module may play a critical role in indirectly regulating linalool biosynthesis. Conversely, the miR167-*Cca.gene21941* (*GPPS*) module may directly regulate monoterpene biosynthesis in linalool-type *C. camphora*. Additionally, miR5368 was found to target Cca.gene21642 (DHDDS) and Cca.gene34720 (GGDR), both of which may contribute directly to linalool biosynthesis.

**Conclusion:**

These findings enhance the understanding of post-transcriptional regulation in linalool biosynthesis and provide insights for developing genetic improvement strategies for *C. camphora*.

**Supplementary Information:**

The online version contains supplementary material available at 10.1186/s12870-025-07588-2.

## Background

*Cinnamomum camphora*, belonging to the Lauraceae family, is an evergreen tree native to China. It serves as both an ornamental plant and a valuable source of timber and aromatic oils, contributing significantly to ecological conservation and economic development [1, 2]. *C*. *camphora* exhibits at least five chemotypes (borneol, linalool, camphor, cineol, and nerolidol) based on the signature constituents in their leaves [3, 4]. The terpenoids extracted from camphor essential oil have widespread applications in the pharmaceutical, chemical, and fragrance industries. Linalool (C_10_H_18_O), a colorless, transparent monoterpenoid liquid, is extensively used in perfumes, cosmetics, household detergents, food, and flavoring agents. In the linalool-type *C*. *camphora*, the linalool content in leaf essential oil can exceed 85%. Compared to other linalool-producing plants, such as coriander, lavender, white orchid, and *Lonicera*, *C. camphora* offers considerable advantages, including higher linalool yield, adaptability to diverse environments, tolerance to poor soils, and substantial biomass production. Furthermore, its suitability for cultivation in dwarf forests and its capacity for continuous yields over multiple years from a single planting. These attributes position *C. camphora* as one of the most promising plant sources for linalool extraction.

The biosynthesis and accumulation of plant secondary metabolites are governed by complex biosynthetic pathways [5, 6]. Plant terpenoids are synthesized through the polymerization of isoprene (C₅) units, forming structures such as monoterpenes (C₁₀), sesquiterpenes (C₁₅), diterpenes (C₂₀), triterpenes (C₃₀), and tetraterpenes (C₄₀). The C₅ precursors, isopentenyl diphosphate (IPP) and its isomer dimethylallyl diphosphate (DMAPP), are produced via two independent pathways: the methylerythritol 4-phosphate (MEP) pathway and the mevalonic acid (MVA) pathway [7]. The MEP pathway utilizes pyruvate and glyceraldehyde 3-phosphate as initial substrates, generating IPP and DMAPP through a series of enzymatic reactions catalysis by DXS, DXR, MCT, CMK, MDS, HDS, and HDR enzymes. The IPP and DMAPP derived from this pathway are primarily utilized for the synthesis of hemiterpenes, monoterpenes, diterpenes, cytokinins, gibberellins, chlorophyll, and plastoquinones [7, 8]. Geranyl diphosphate (GPP), synthesized from one molecule of IPP and one molecule of DMAPP geranyl diphosphate synthase (GPPS), serves as the direct precursor for the monoterpene, including linalool. Furthermore, IPP can undergo sequential condensation with DMAPP to form longer-chain precursors: farnesyl diphosphate (FPP, C₁₅; precursor to sesquiterpenes) and geranylgeranyl diphosphate (GGPP, C₂₀; precursor to diterpenes and polyterpenes). Linalool is produced in a single enzymatic step catalyzed by linalool synthase (*TPS*/*LIS*) using GPP as a substrate. Consistent with this pathway, our previous study demonstrated that overexpression of the *C. camphora* linalool synthase gene *CcTPS5a* in *Arabidopsis thaliana* led to increased linalool accumulation.

MicroRNAs (miRNAs) are a class of endogenous, non-coding and single-stranded RNAs, typically 20–24 nucleotides in length, that play critical role in post-transcriptional regulation. They are involved in plant growth, metabolism, and responses to abiotic and biotic stress [9–14]. An increasing number of studies have indicated that miRNAs modulate terpenoid biosynthesis by inhibiting the expression of transcription factors or by directly targeting structural genes within the terpenoid synthesis pathway. For instance, miR156 influences sesquiterpene biosynthesis by targeting SQUAMOSA PROMOTER-BINDING PROTEIN-LIKE 9 (SPL9) [15]. In *Camellia sinensis*, miR156-SPL mediates the synthesis of terpenoids [16]. In *Ginkgo biloba*, miR167, miR163c, and miR160 participate in the biosynthesis of terpene trilactones by targeting *ACAT* (acetyl-CoA acetyltransferase), *GGPPS* (geranylgeranyl diphosphate synthase), and GbERF4 (an ERF transcription factor), respectively [17]. Advances in high-throughput sequencing have accelerated the identification of miRNAs regulating terpenoid biosynthesis in various species [18]. For example, miR5021, miR1134, and miR7539 are involved in terpenoid biosynthesis in *Xanthium* by targeting *IDS*, *DXS*, *IDI*, and *HMGR* [19]. Additionally, miR5021 has been reported to regulate terpenoid biosynthesis in *Podophylum*, *Vinca*, and *Mentha* by targeting *IDI*, *GGPP*, *GGPS*, and other related genes [20–22]. In our previous work, using high-throughput sequencing without a reference genome, we identified several miRNAs (e.g., miR5021 target GGDR, miR4995 target indole-3-pyruvate monooxygenase,) and genes associated with terpenoid synthesis in different chemotypes of camphor trees [2, 23]. Reports on miRNA-mediated regulation of linalool biosynthesis remain limited. In the tea plant (*C*. *sinensis*), linalool content shows a strong positive correlation with the expression levels of miR171o and miRN71 [24]. In *Hedychium coronarium*, knockdown of hco-miR167n and hco-miR393a using short tandem target mimic (STTM) technology increases linalool accumulation. Similarly, VIGS-mediated silencing of *HcARF8* and *HcTIR1* also significantly enhances linalool levels [25]. Despite sporadic reports of miRNAs involvement in linalool biosynthesis in other species, no systematic research has been conducted in *C. camphora* (a crucial source plant of linalool). The identities of miRNAs and their target genes involved in linalool biosynthesis in *C. camphora*, as well as the mechanisms underlying their regulatory effects, have not been elucidated.

In this study, we measured the total linalool content of 20 linalool-type *C. camphora* trees in March and May, categorizing them into three groups on the basis of high, medium, and low linalool groups. High-throughput sequencing was performed on leaves with a high linalool content collected in March (H_MAR) and May (H_MAY), as well as leaves with a low linalool content from May (L_MAY). Our objectives were to dentify key miRNAs and their target genes and to construct a regulatory network underlying miRNA-mediated linalool synthesis. The findings of this study provide a valuable foundation for future efforts to enhance linalool content and improve camphor varieties through genetic engineering.

## Results

### Trends in linalool content

**Fig. 1:**
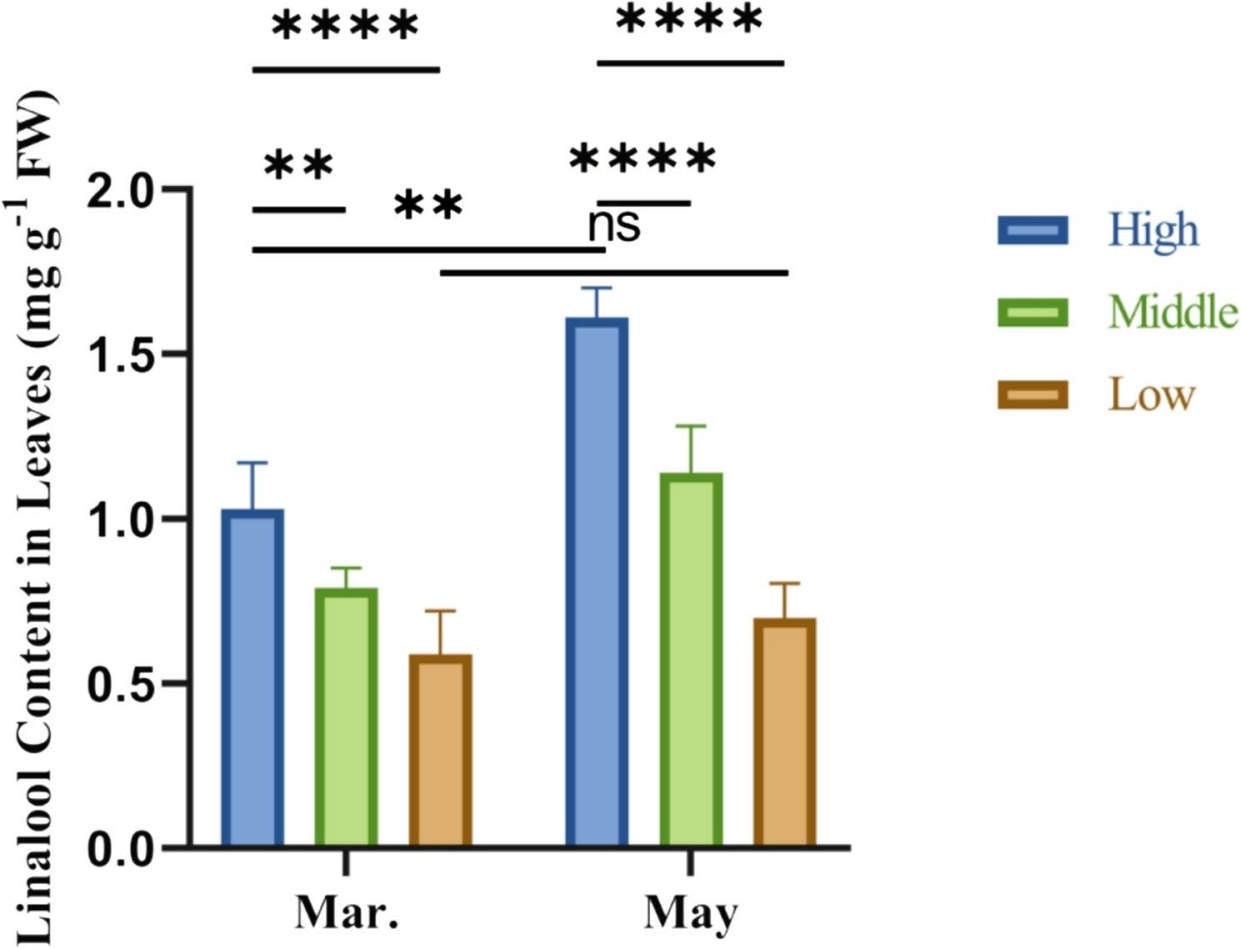
Linalool content obtained using GC-MS and water distillation from linalool chemotype leaves of *C. camphora*. FW: fresh weight

The linalool content was calculated based on the proportion of linalool measured by GC-MS and the total essential oil yield (Fig. 1). Leaves collected in May were categorized into three groups based on linalool content: low (< 0.9 mg g⁻¹ FW), medium (0.9–1.5 mg g⁻¹ FW), and high (> 1.5 mg g⁻¹ FW). Significant differences in linalool content were observed among the three groups within the same month. In the high-content group, linalool levels increased significantly in May compared to March, whereas no significant changes were detected in the low-content group.

### Small RNA analysis and miRNA identification

**Fig. 2:**
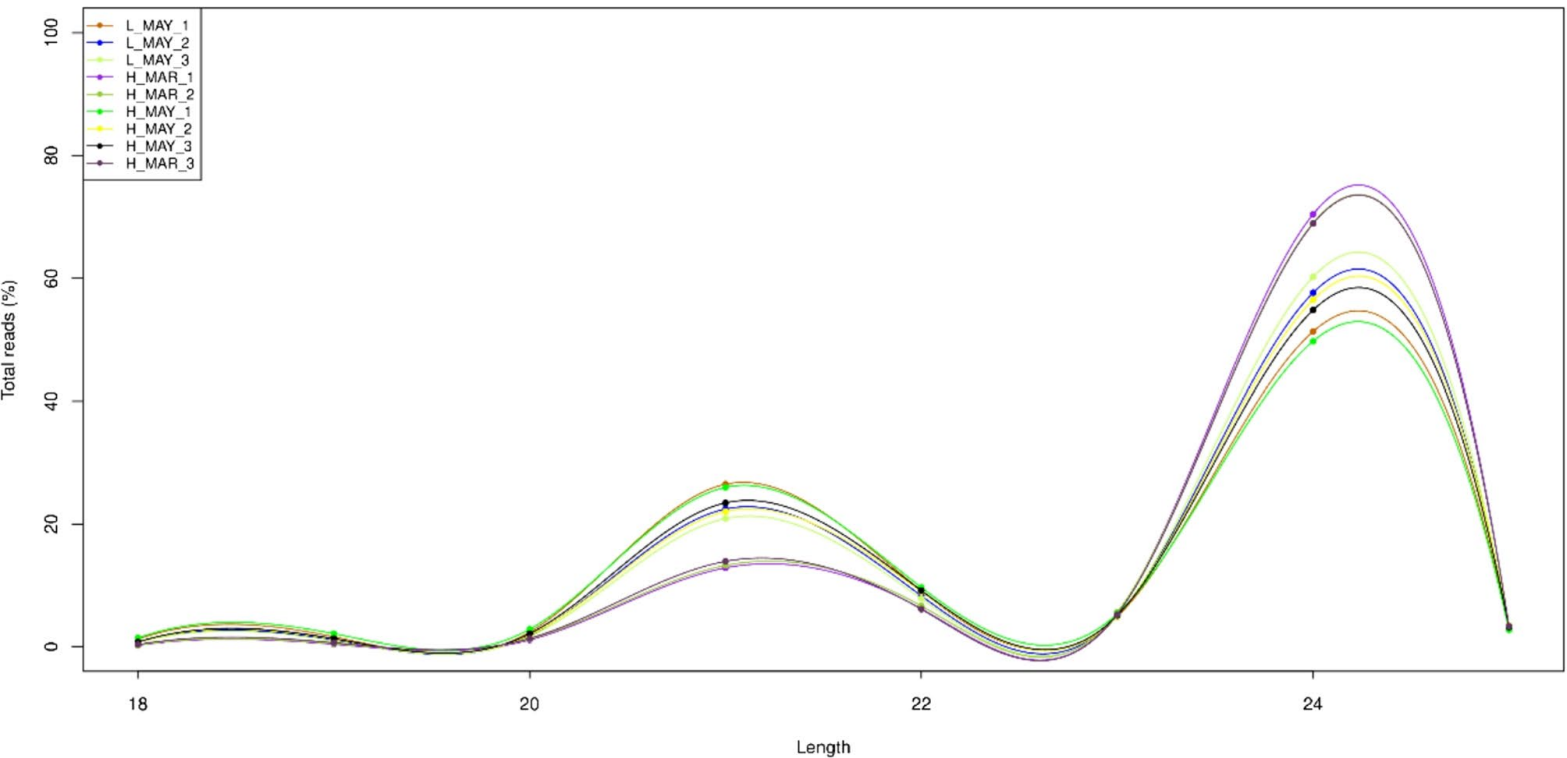
Length distribution of the small RNAs

Small RNA sequencing of the nine libraries (L_MAY_1–3, H_MAR_1–3, H_MAY_1–3) generated between 9,108,546 and 12,326,213 reads per library (Table S2). The Phred quality score at Q20 and Q30 ranged from 98.34% to 98.72% and 95.09% to 95.97%, respectively. The GC content of the nine libraries was between 51.56% and 52.88%. After removal of junk reads, 3ADT&length filter reads, and non-miRNA sequences mapped to Repbase and the Rfam database, valid reads were retained for further analysis. Reads between 18 and 25 nucleotides in length were retained, resulting in final datasets containing 6,856,572 to 9,927,850 sequences per library. Statistical analysis revealed that 24-nucleotide sequences were the most abundant across all nine libraries, followed by 21-nucleotide sequences (Fig. 2).

### Identification of miRNAs in *C*. *camphora*

**Fig. 3:**
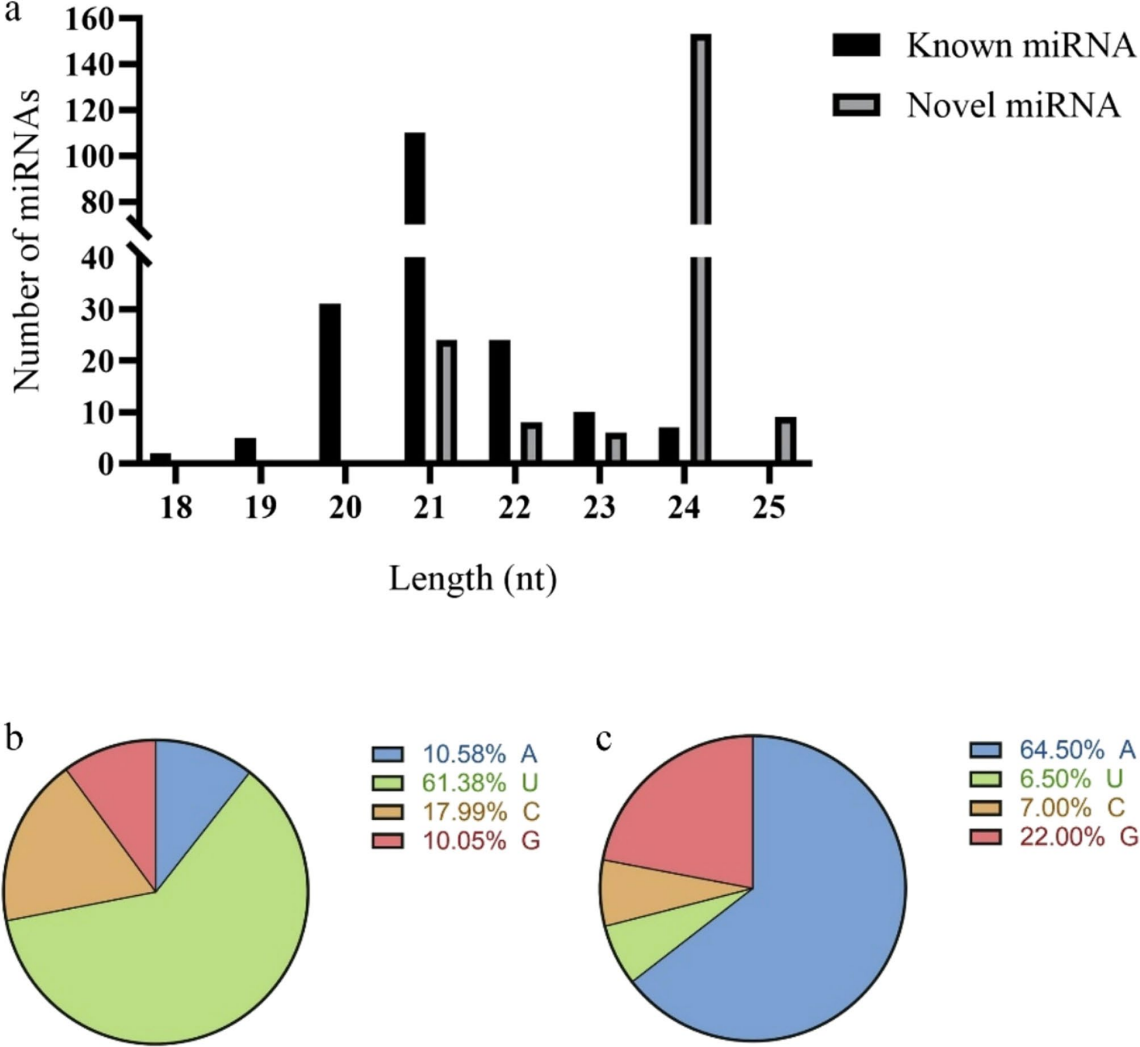
Length distribution of identified miRNAs and the first position nucleotide bias of miRNAs. **a** A total of 399 miRNAs were found and divided into two classes: known miRNAs and novel miRNAs. **b** The first-position nucleotide bias in known miRNAs. **c** The first-position nucleotide bias in novel miRNAs

A total of 399 miRNAs were identified across the H_MAR, H_MAY, and L_MAY samples (Table S3). Among these, 199 were known miRNAs that matched mature miRNA sequences in miRBase v. 22.0, and 200 were novel miRNAs, designated as predicted candidates (PCs), which mapped to the genome but not to known miRNAs in miRBase (Table S4). Among the known miRNAs, 21-nucleotide sequences were the most abundant, followed by 24-nucleotide sequences. In contrast, for the novel miRNAs, 24-nucleotide sequences were the most abundant, followed by 21-nucleotide sequences (Fig. 3a), a distribution consistent with our previous report [2]. Sequence analysis revealed a strong bias toward uracil (U) at the first nucleotide position for known miRNAs (Fig. 3b), which aligns with patterns observed in most plants. Novel miRNAs showed a bias toward adenine (A) at the first nucleotide (Fig. 3c).

Conservation analysis of known miRNAs indicated that the miR156 family had the largest number of members (15), followed by miR166 (14) and miR159 (13) (Table S5). Most of these miRNAs exhibited high homology miRNAs from *Glycine max*, *Malus domestica*, and *Populus trichocarpa* (Table S6, Fig. S1), suggesting conserved functional roles in plant biological processes.

#### DEM identification and validation

**Fig. 4:**
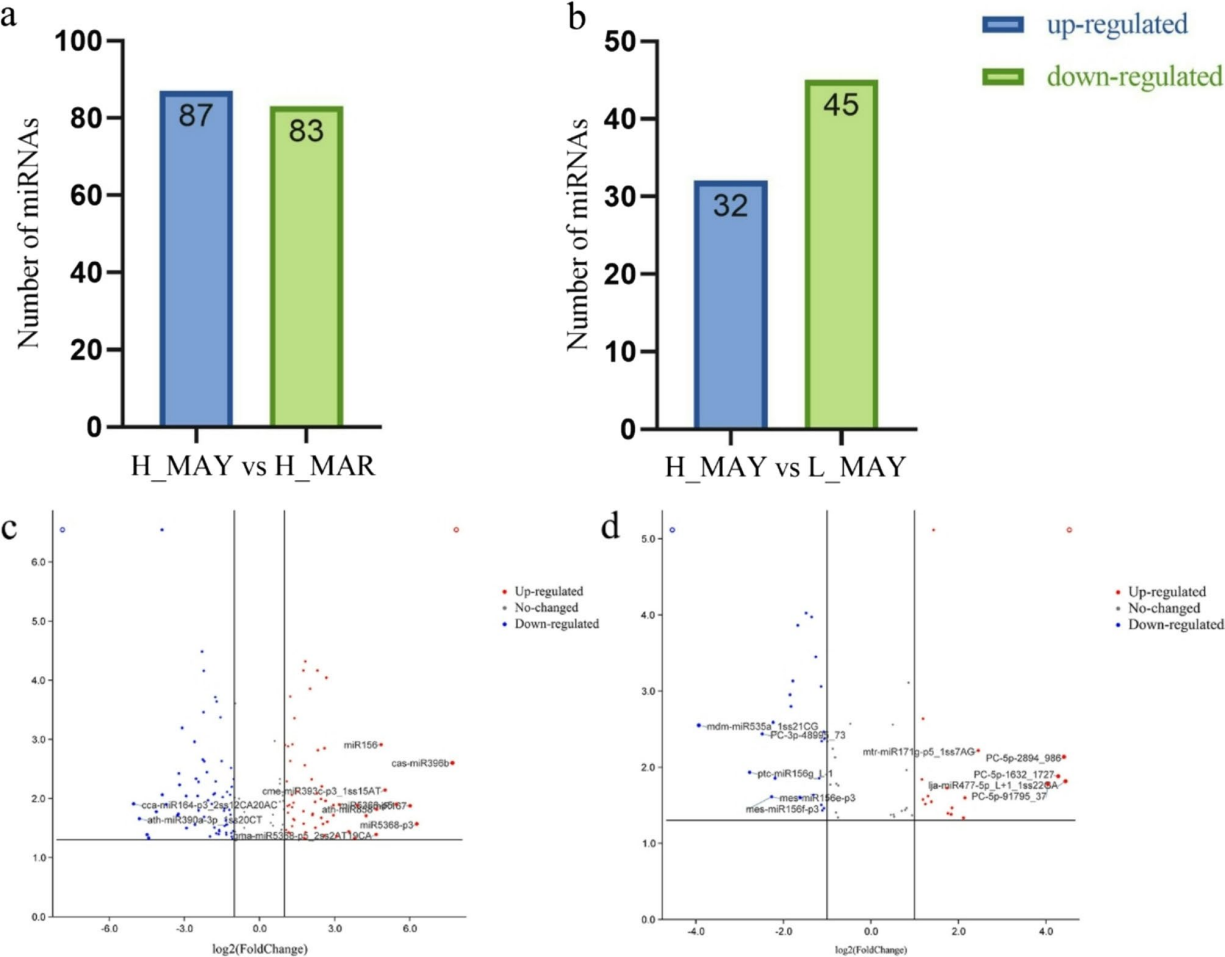
Differentially expressed miRNAs in different groups. **a** The number of DEMs between H_MAY and H_MAR. **b** The number of DEMs in H_MAY and L_MAY. **c** The datasets generated or analysed during the current study are available in the supplementary materials and Genome Sequence Archive repository, GSA: CRA025779.) the volcano of DMEs between H_MAY and H_MAR. d, the volcano of DMEs between H_MAY and L_MAY

Based on their expression levels, the 399 miRNAs were classified into high- (*n* = 65), medium- (*n* = 294), and low-expression (*n* = 40) groups (Table S7). Differential expression analysis identified 212 differentially expressed miRNAs (DEMs) across the sample groups (Table S8). A greater number of DEMs was detected between H_MAY and H_MAR than between H_MAY and L_MAY. Specifically, 170 DEMs (83 downregulated and 87 upregulated) were identified in the H_MAY vs. H_MAR comparison (Fig. 4a, Fig. S2), whereas 77 DEMs (45 downregulated and 32 upregulated) were found in the H_MAY vs. L_MAY comparison (Fig. 4b, Fig. S3).

To visually expression trends of DEMs potentially involved in linalool biosynthesis, we selected the top 10 DEMs from different comparison groups for further examination. The expression levels of miR156, miR393, miR396b, miR858, and miR5368 were significantly higher in H_MAY than in H_MAR, whereas miR164 and miR390 were significantly lower (Fig. 4c). In the H_MAY vs. L_MAY comparison, the expression levels of miR535, miR156, and PC-3p-48995_73 were significantly lower in H_MAY, while miR171, miR477, PC-5p-2894_986, PC-5p-1632_1727, and PC-5p-91795_37 were significantly higher (Fig. 4d). These miRNAs may play potential regulatory roles in linalool biosynthesis, and their functions were further investigated alongside degradome sequencing data.

**Fig. 5:**
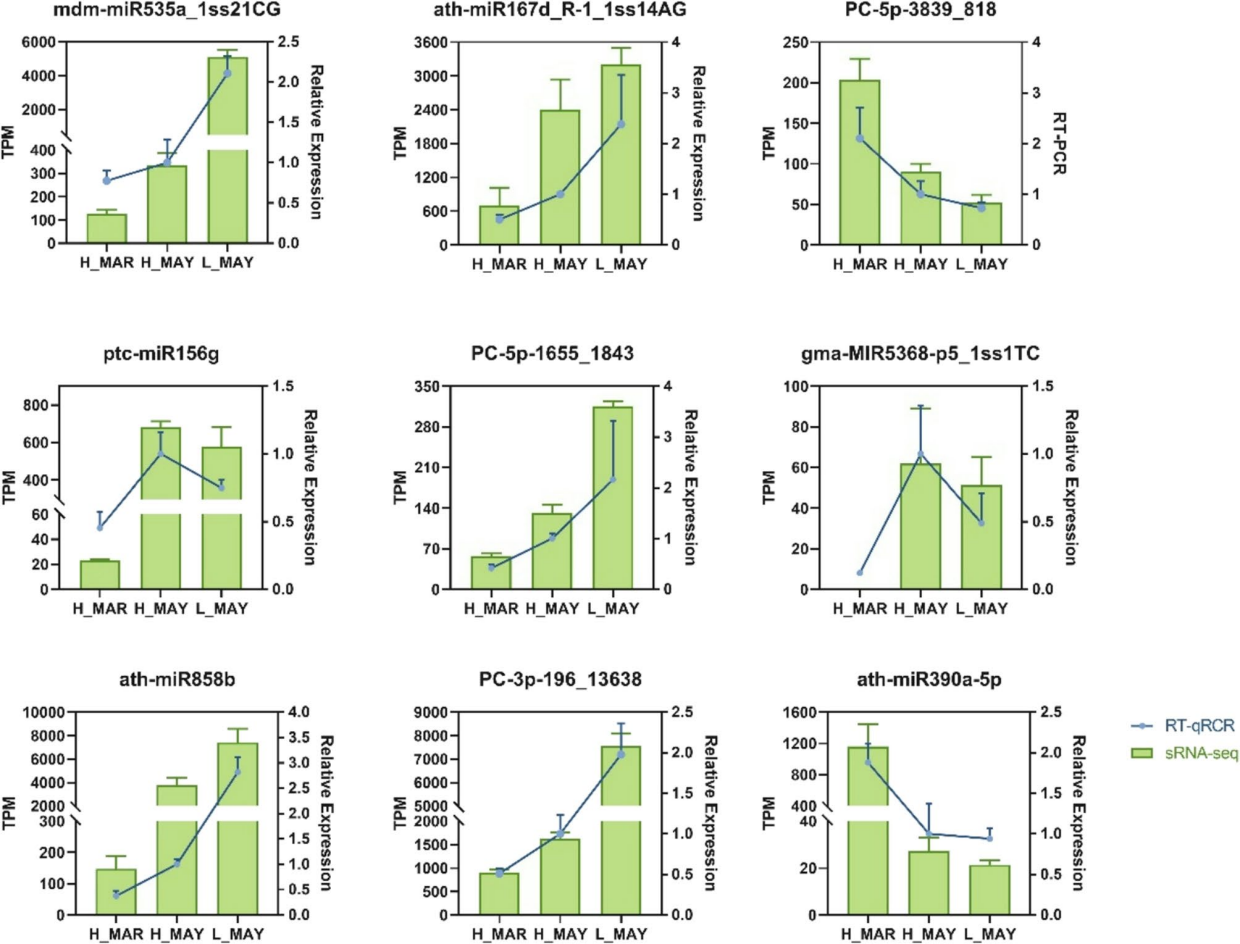
Validation of miRNA quantification. Comparison and correlation analysis of nine miRNAs. The X-axis represents the three samples of leaves: H_MAR, H_MAY, and L_MAY. The Y-axis represents the relative expression level and transcripts per million

Nine DEMs were randomly selected to validate the accuracy of miRNA sequencing (Fig. 5). The expression profiles obtained from RT-qPCR were consistent with the TPM values from sequencing, confirming the reliability of small RNA the sequencing data.

### Target gene prediction by using degradome data

Degradome sequencing was used to eliminate false-positive target genes lacking mRNA cleavage sites. A total of 12,072,197 raw reads and 5,672,718 unique reads were generated (Table 1). After the removal of short reads (< 15 nucleotides) and 3′ adaptors, 23,827 transcripts were retained and analyzed for target prediction by using CleaveLand v. 4.0.

**Table 1:** Degradome sequencing data of *C*. *camphora*

Sample	Number	Ratio
Raw Reads	12,072,197	/
Unique Raw Reads	5,672,178	/
Reads < 15 nt (after removing 3’ adaptor)	100,285	0.83%
Mappable Reads	11,971,912	99.17%
Unique reads < 15 nt (after removing 3’adaptor)	41,005	0.72%
Unique Mappable Reads	5,631,173	99.28%
Mapped Reads	6,773,461	56.11%
Unique Mapped Reads	2,626,853	46.31%
Number of Input Transcripts	36,411	/
Number of Covered Transcripts	23,827	65.44%

In total, 375 genes were targeted by 107 miRNAs, forming 567 miRNA-target pairs (Table S9). These pairs were categorized into five groups (0, 1, 2, 3, 4) based on the relative abundance of cleavage signals [26]. Categories 0, 1, and 2 (comprising 95, 2, and 181 pairs, respectively) represent the most reliable targets, while 55 and 234 pairs fell into Categories 3 and 4, respectively. Among the DEMs, degradome sequencing identified 309 target genes regulated by 91 DEMs, resulting in 492 miRNA-target pairs. This included 223 targets associated with 52 known miRNAs and 86 targets of 39 novel miRNAs. A total of 435 miRNA-target pairs were identified from H_MAY vs. H_MAR comparison, and 123 pairs from the H_MAY vs. L_MAY comparison.

### GO and KEGG analyses of target genes

**Fig. 6:**
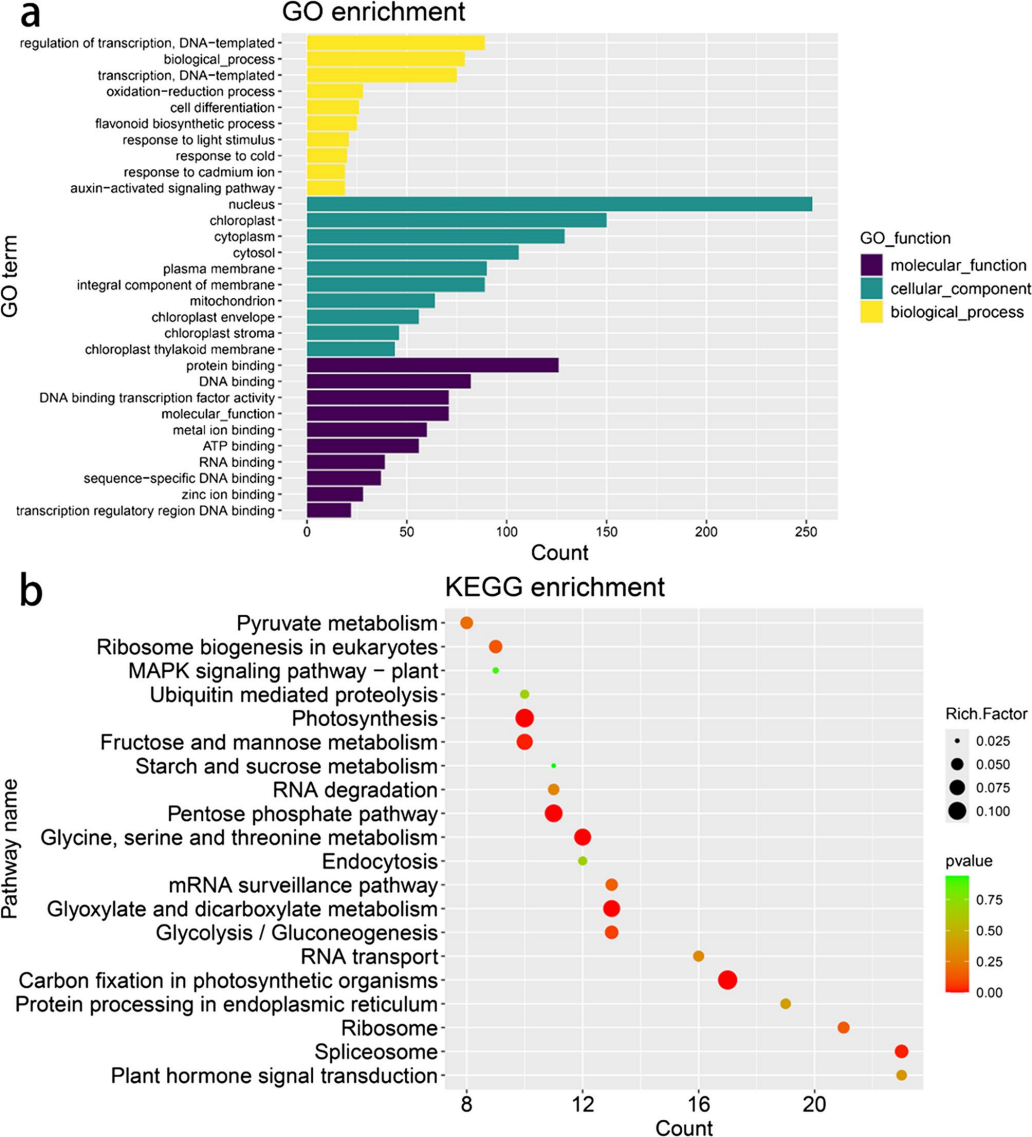
Annotation information for targets of miRNAs in *C*.*Camphora*. **a** Scatter diagram of the enrichment of target genes in GO terms. Different colors denote different GO categories. **b** KEGG annotation of the target genes. The left coordinate represents the terms or pathways, and the horizontal coordinate represents the total number of target genes in terms or pathways. The plot size represents the enrichment factor, and the *p*-value was obtained using a hypergeometric test

Gene Ontology (GO) analysis identified 357 target genes, categorized into the biological process (BP), molecular function (MF), and cellular component (CC) domains. The top 10 terms in each category are shown in Fig. 6a. In the BP category, the most enriched terms were “regulation of transcription, DNA-templated,” “biological process,” and “transcription, DNA-templated.” For CC, the target genes were primarily associated with the “nucleus,” “chloroplast,” and “cytoplasm.” In the MF category, “protein binding” and “DNA binding” were the most highly represented terms.

Kyoto Encyclopedia of Genes and Genomes (KEGG) pathway analysis annotated 158 target genes to 103 pathways, with the top 20 pathways selected for further analysis (Fig. 6b). The most enriched pathways included “Spliceosome” (ko03040), “Plant hormone signal transduction” (ko04075), and “Ribosome” (ko03010) (Fig. 6b). Among the targets of DEMs, *Cca.gene21642* (dehydrodolichyl diphosphate synthase 2-like protein, DHDDS), *Cca.gene21941* (heterodimeric geranyl diphosphate synthase small subunit protein 1, GPPS), and *Cca.gene34720* (geranylgeranyl diphosphate reductase, GGDR) were annotated to the “Terpenoid backbone biosynthesis” pathway (ko00900), these genes are directly involved in the terpenoid biosynthesis process. Furthermore, transcription factors including AP2/ERF (*Cca.gene10826*), bHLH (*Cca.gene39046*), and SPL (*Cca.gene5634*, *Cca.gene124*, *Cca.gene16950*, *Cca.gene38360*, *Cca.gene7184*, *Cca.gene9662*) were significantly enriched. These regulators may indirectly modulate terpenoid biosynthesis by controlling the expression of key genes involved in the terpenoid synthesis pathway.

### MiRNA and target gene regulatory network analysis

**Fig. 7:**
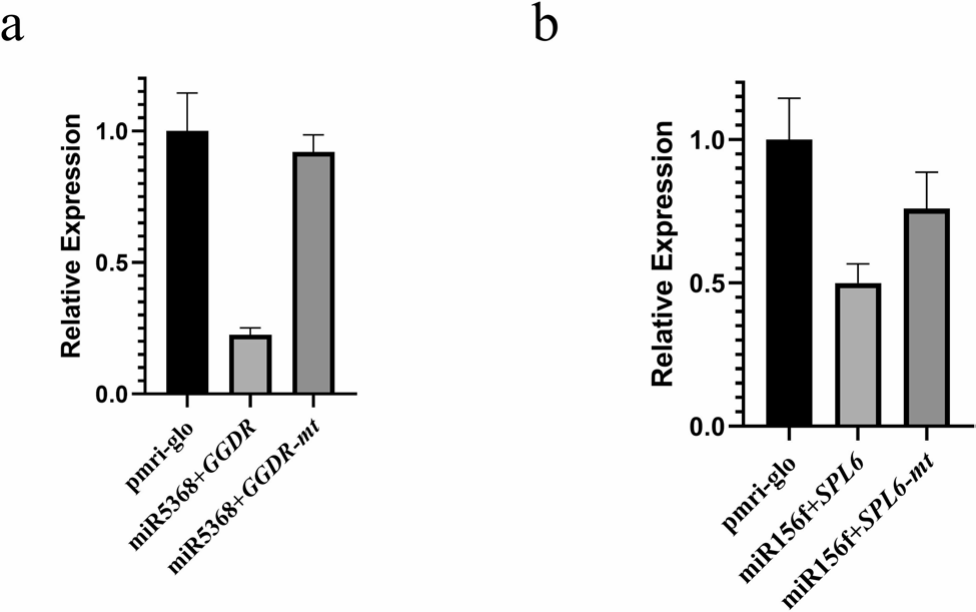
The result of dual-luciferase reporter assay. **a** The results of the dual luciferase reporter system between miR5368 and *GGDR*. **b** The results of the dual luciferase reporter system between miR156 and *SPL6*

By integrating small RNA and degradome sequencing results, we constructed a regulatory network of DEMs and their target genes (Figs. S4 and 7) and superimposed these interactions onto a terpenoid biosynthesis pathway map (Fig. 9). To validate the degradome sequencing results, we analyzed the TPM values for five miRNA-target pairs, which revealed complementary expression patterns between the miRNAs and their target genes, providing preliminary confirmation of their regulatory relationships (Fig. S5). The analysis highlighted miRNAs that target key structural genes within the terpenoid biosynthesis pathway, suggesting direct regulatory roles in linalool synthesis. For example, *Cca.gene21642* (*DHDDS*) and *Cca.gene34720* (*GGDR*) were both targeted by gma-miR5368-p5_1ss1TC, which exhibited significantly higher expression in H_MAY than in H_MAR. To further investigate the regulatory relationship between miR5368 and its putative target gene *GGDR*, a dual-luciferase reporter assay was conducted. The results indicated that the relative luminescence signal of the wild-type target group (pmriGLO:*GGDR*) was significantly reduced to 22.57%, whereas that of the mutated target group (pmriGLO:*GGDR*-*mt*) recovered to 92.09% compared to the control (Fig. 7a). This suggests that gma-miR5368-p5_1ss1TC may regulate linalool synthesis by targeting these mRNAs. Furthermore, *Cca.gene21941* (*GPS*) was identified as a target of mdm-miR167h_R-1_1ss19CT. Although the expression level of mdm-miR167h_R-1_1ss19CT was significantly higher in H_MAY than in H_MAR. However, this expression pattern does not align with the observed linalool content differences between these groups. We hypothesize that mdm-miR167h_R-1_1ss19CT may participate in the early stages of linalool synthesis and accumulation.

The results also indicated that miRNA may indirectly affect terpenoid biosynthesis by targeting transcription factors. Several SPL family members (*SPL3*, *SLP6*, *SLP13A*, *SPL16*, and *SPL17)* were identified as targets of miR157a (aly-miR157a-5p_R + 2 and ath-miR157a-5p_R + 1), miR156a/f/g/l (bra-miR156a-5p, ppe-miR156f_L + 1, ptc-miR156g and ptc-miR156g_L-1), and miR535a (mdm-miR535a and mdm-miR535a_1ss21CT). The miR156-SPL module has been widely reported to regulate terpenoid biosynthesis across various plant species. To validate the accuracy of our degradome sequencing results, the regulatory relationship between ppe-miR156f_L + 1 and *Cca.gene38630* (*SPL6*) was investigated using a dual-luciferase reporter assay. The results demonstrated that the relative luminescence signal of the wild-type target group was significantly reduced to 49.92%, whereas that of the mutated target group recovered to 75.99% compared with the control (Fig. 7b). These results underscored the regulatory role of the miRNA-*SPL* module in terpenoid biosynthesis and its potential involvement in linalool production. A comprehensive analysis integrating miRNA expression and linalool content revealed that the complexity of this regulatory module. In the H_MAY vs. H_MAR comparison, several miRNAs targeting SPLs (e.g., ath-miR157a-5p_R + 1, bra-miR156a-5p, ppe-miR156f_L + 1, ptc-miR156g, mdm-miR535a) were significantly upregulated in H_MAY. Conversely, in the H_MAY vs. L_MAY comparison, bra-miR156a-5p, mdm-miR535a, and mdm-miR535a_1ss21CT were significantly more highly expressed in L_MAY. We propose that bra-miR156a-5p, mdm-miR535a, and mdm-miR535a_1ss21CT not only regulate the early synthesis and accumulation of linalool but also play significant roles in the linalool biosynthesis pathway of both high and low linalool content camphor trees. Further analysis revealed that the bHLH transcription factor gene *Cca.gene39046* (bHLH113) is targeted by PC-3p-100252_33, which showed significantly lower expression in H_MAY than in H_MAR. Similarly, the AP2/ERF transcription factor gene *Cca.gene10826* (AP2/ERF) is regulated by mes-miR156e-p3, which was significantly more highly expressed in L_MAY than in H_MAY. The expression patterns of both PC-3p-100252_33 and mes-miR156e-p3 correlate with the observed trends in linalool content trends, suggesting that these miRNA-transcription factor modules are involved in the regulation of terpenoid and linalool biosynthesis.

## Discussion

Our analysis of linalool content in 20 *C. camphora* trees revealed a significant difference in the linalool content among the high, medium, and low groups, and the linalool content significantly increased in the high group from March to May. Multi-omics approaches have increasingly highlighted the role of miRNAs as key post-transcriptional regulators of terpenoid biosynthesis [27, 28]. However, only a few miRNAs in monoterpene biosynthesis have been functionally characterized [29]. Moreover, the function of miRNAs in monoterpene biosynthesis in linalool-type *C. camphora* remains limited. The present study addresses this gap by investigating the miRNA-mediated regulation of linalool biosynthesis in *C. camphora*.

In this study, 199 known miRNAs and 200 novel miRNAs were identified from the three samples differing in linalool content. In our previous study, deep sequencing of linalool- and borneol-type camphor leaves without a reference genome revealed 364 known and 117 novel miRNAs [2]. Hou et al. (2023) identified 876 miRNAs in *C. burmannii* leaves at four developmental stages through small RNA sequencing [30]. Wang et al. (2022) detected 1,563 miRNAs in camphor leaves from five chemotypes by using chromosome-level genome assembly [31]. The number of identified miRNAs varies on the basis of sample size, sample type, developmental stage, and analytical methods. Genome-wide miRNA identification, in particular, significantly increases detection rates. The 399 miRNAs identified in this study not only expand the known repertoire of camphor miRNAs but also contribute to advancing research on miRNA regulation of monoterpene biosynthesis in linalool-type *C. camphora*.

**Fig. 8:**
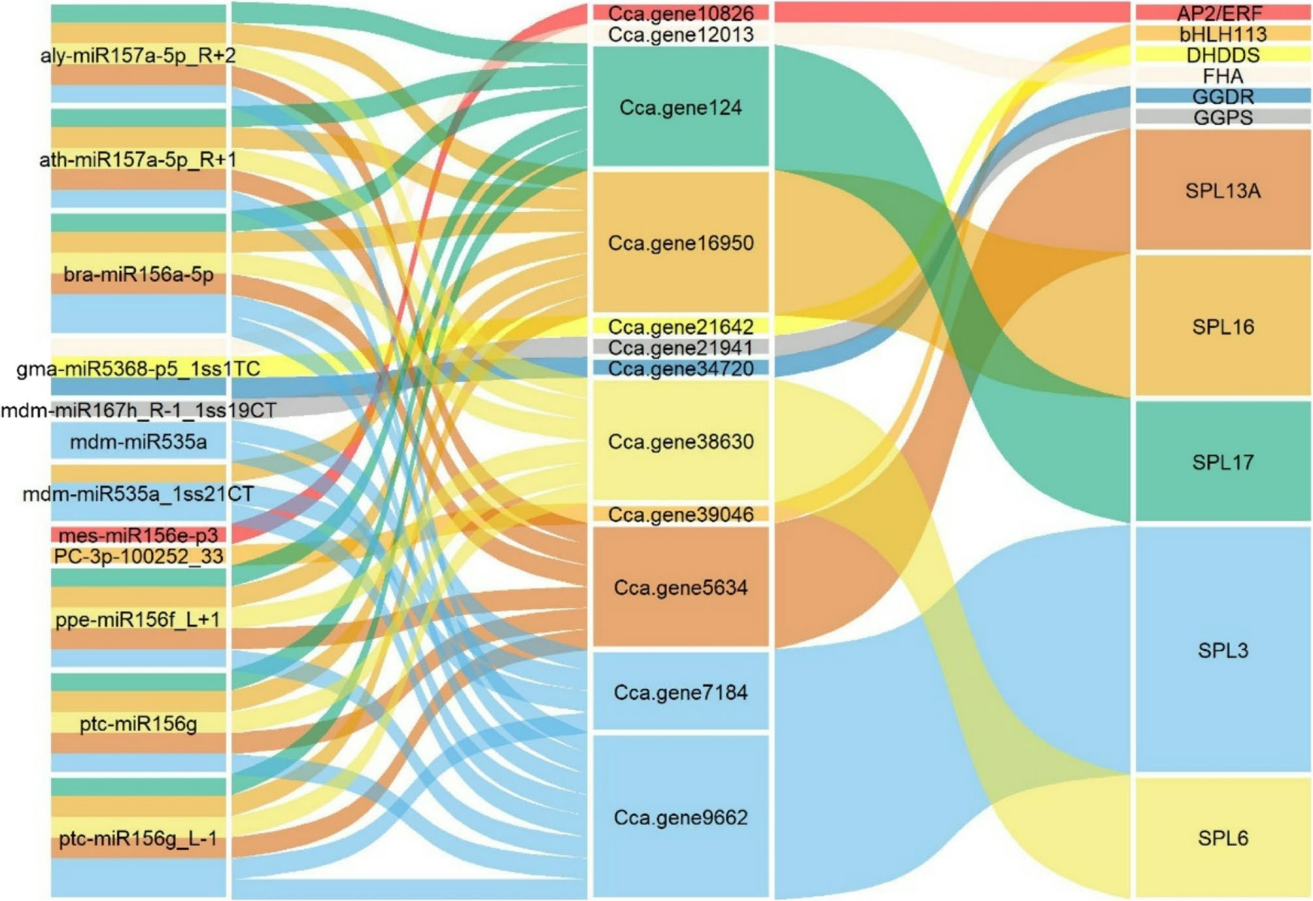
Sankey diagram of miRNA-target modules involved in linalool biosynthesis of *C*. *camphora*. The miRNAs are shown on the left, their target gene IDs are in the middle, and their target genes are listed on the right. Different colors represent different target genes

Unlike enzyme-encoding structural genes, transcription factors (TFs) often regulate terpenoid biosynthesis indirectly. The SPL family of TFs has been implicated in this process in several species. In *Arabidopsis*, the miR156-*SPL* module regulates E-β-caryophyllene biosynthesis by modulating *TPS21* expression, and it also mediates patchouli oil synthesis [15]. In *Dryopteris fragrans*, miR156 regulates terpenoid biosynthesis by targeting *SPL3*, which induces *GGPPS1* expression [32]. In addition, the miR156-*SPL* module mediates terpenoid synthesis in *C. sinensis* [16]. In the present study, miR157a (aly-miR157a-5p_R + 2 and ath-miR157a-5p_R + 1), miR156a/f/g (bra-miR156a-5p, ppe-miR156f_L + 1, ptc-miR156g, and ptc-miR156g_L-1), and miR535a (mdm-miR535a and mdm-miR535a_1ss21CT) were found to target *SPL3*, *SPL6*, *SPL13A*, *SPL16*, and *SPL17* (Figs. 7 and 8), suggesting that a conserved miRNA-SPL module may regulate terpenoid biosynthesis in *C*. *camphora*, potentially influencing linalool production through a miRNA-SPL-TPS network. Concurrently, the study revealed that the miRNA-SPL module mediating linalool biosynthesis represents a complex regulatory process. The expression patterns of bra-miR156a-5p, mdm-miR535a, and mdm-miR535a_1ss21CT across different comparisons indicate their potential involvement in both the initiation and maintenance of linalool biosynthesis in trees with high and low linalool yields, although these proposed functions require further experimental validation.

Other TFs, including AP2/ERF and bHLH, are known to regulate terpenoid synthesis. Among them, *Arabidopsis* AtMYC2 [33, 34]、Wintersweet *CpbHLH13* [35], *Osmanthus bHLH35* [36], and peach *PpERF5*/*7*/*61* [37] are involved in linalool biosynthesis by regulating *TPS* genes. In the present study, degradome sequencing predicted that PC-3p-100252_33 targeted *Cca*.*gene39046* (*bHLH113*) and mes-miR156e-p3 targeted *Cca.gene10826* (*AP2/ERF*) (Figs. 7 and 8), implicating them in linalool biosynthesis. The correlation between the expression patterns of these miRNAs and linalool content supports this hypothesis, but functional studies are needed to confirm their roles.

**Fig. 9:**
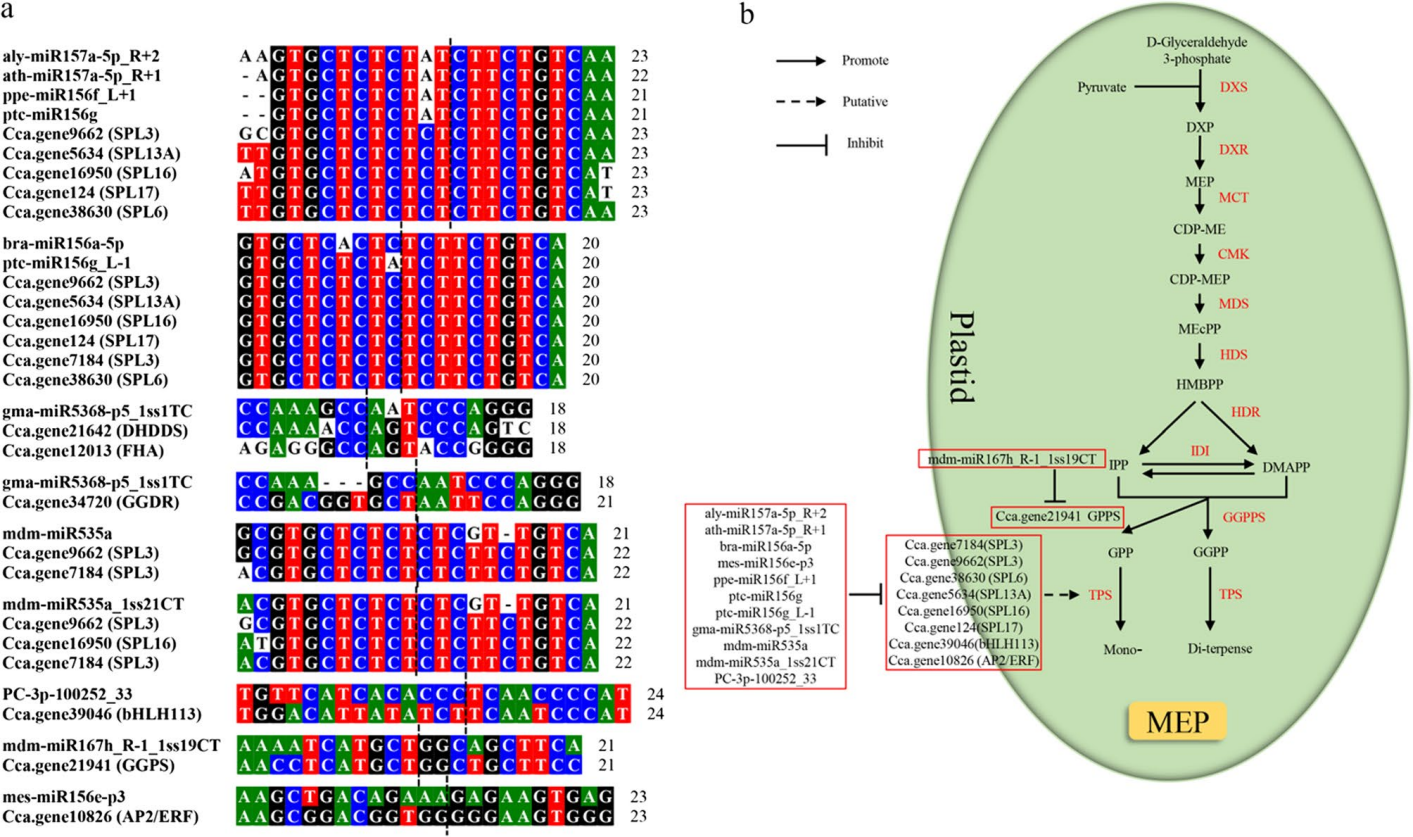
MicroRNA-mediated linalool biosynthesis. **a** Alignment of miRNAs and the miRNA response element (MRE) of target genes, the dotted line indicates the cleavage site. **b** Enzymes involved in terpenoid biosynthesis are marked in red fonts, whereas miRNAs and target genes involved in linalool biosynthesis are listed in red boxes

While the core genes of terpenoid biosynthesis are conserved in plants, the miRNAs target them can be species-specific [38, 39]. MiRNAs such as miR396/6300-*HMGS* [14, 40], miR1134/5021/6300-*HMGR* [19, 40], miR530/5021-*MVD* [20, 40], as well as several novel miRNAs have been predicted in various plants. And the miR5021-*HMGR* interaction, which regulates the rate-limiting step of the mevalonate pathway, is among the few conserved interactions across some plant species [16, 38, 39, 41]. In this study, we identified three key terpenoid biosynthesis enzymes, namely *Cca.gene21642* (*DHDDS*), *Cca.gene34720* (*GGDR*), and *Cca.gene21941* (*GGPS*), were targeted by gma-miR5368-p5_1ss1TC and mdm-miR167h_R-1_1ss19CT, forming the gma-miR5368-p5_1ss1TC-*Cca.gene21642* (*DHDDS*)/*Cca.gene34720* (*GGDR*) and mdm-miR167h_R-1_1ss19CT-*Cca.gene21941* (*GPPS*) pairs (Fig. 8). We previously reported miR5021 targets *CcGGDR*, which is the backbone gene of the diterpenoid pathway that uses GGPP as a substrate and may compete for the common substrate GPP of monoterpenoid linalool biosynthesis [2]; thus, miR5368 might function similarly. In most plants, terpene precursors, such as GPP, are typically synthesized in plastids through *GPPS* via the methylerythritol phosphate pathway. Interestingly, in *C. camphora*, GPP biosynthesis is predominantly governed by the MVA pathway, with *GPPS* and *TPS* catalyzing catalyzing monoterpenoid formation. While miR5015 targets *GPPS* in rose-scented geranium [42] and miR164-3p targets *RrGPPS* in rose [13], we identified miR167 as a DEM targeting * *Cca.gene21941* (*GPPS*), suggesting its specific involvement in linalool synthesis in *C*. *camphora* (Fig. 9b). This miRNA-gene pair may underlie differences in linalool among the H_MAY, H_MAR, and L_MAY samples, playing a key role in monoterpene biosynthesis in linalool-type *C*. *camphora*. Future experimental validation is essential to confirm the functions of these candidate miRNAs and their targets.

## Conclusion

Integrating small RNA and degradome sequencing, we systematically analyzed the post-transcriptional regulation of linalool biosynthesis pathways in the L_MAY, H_MAY, and H_MAR samples with varied linalool contents. A total of 199 known miRNAs and 200 novel miRNAs were identified, with degradome sequencing predicting 375 target genes regulated by 107 miRNAs. Network analysis of DEMs and their targets suggested that the miRNA-*SPL* module may indirectly regulate linalool biosynthesis. Furthermore, miR167 targeted *Cca.gene21941* (*GPPS*), whereas miR5368 targeted *Cca.gene21642* (*DHDDS*) and *Cca.gene34720* (*GGDR*), that appear to directly modulate monoterpene biosynthesis in linalool-type *C. camphora*. These findings advance our understanding of post-transcriptional regulatory mechanisms controlling linalool biosynthesis and provide valuable insights for future genetic improvement of camphor varieties.

## Materials and methods

### Measurement of Linalool content

Fresh leaves were from 20 linalool-type *C. camphora* trees were collected in March and May 2019 at the experimental tree farm of the Jiangxi Academy of Forestry in Nanchang, China for essential oil extraction and analysis. Leaves were sampled from the lower apical portions of one-year-old branches located on the sun-exposed side of ten-year-old trees. Essential oils were individually extracted from each tree using hydrodistillation with a modified Clevenger-type apparatus, and terpene composition was analyzed through gas chromatography-mass spectrometry (GC-MS). The detailed protocol has been described in our previous study [23].

### RNA isolation

Based on linalool content analysis, leaves with the highest linalool level were collected in March (H_MAR_1, 2, 3) and May (H_MAY_1, 2, 3), whereas those with the lowest content were sampled in May (L_MAY_1, 2, 3). Immediately after collection, all leaves were flash-frozen in liquid nitrogen and stored at − 80 °C. Total RNA from nine samples was extracted using TRIzol reagent (Invitrogen, CA, USA) in accordance with the manufacturer’s protocol. RNA quantity and purity were determined using a Bioanalyzer 2100 and an RNA 6000 Nano LabChip Kit (Agilent, CA, USA), ensuring an RNA integrity number of ≥ 7.

### Library construction and small RNA-seq

A total of 1 µg of RNA was used to construct a small RNA library following the TruSeq Small RNA Sample Prep Kit protocol (Illumina, San Diego, USA). After the quality assessment, all nine libraries underwent single-end sequencing (1 × 50 bp) on an Illumina HiSeq 2500 (Illumina, San Digeo, CA, USA) at Lianchuan Biotechnology Co., Ltd., China, in accordance with the vendor’s recommended procedures.

### Small RNA data analysis and miRNA identification

Raw data were processed using the in-house program ACGT101-miR (v4.2) (LC Sciences, Houston, Texas, USA) to remove poly-N (N percentage >10%), 3’ adapter null reads, 5’ adapter-contaminated reads, low-complexity reads, and poly-T/A/C/G reads. Sequences shorter than 18 nucleotides or longer than 25 nucleotides were also filtered out. Unique sequences with a length of 18–25 nucleotides were aligned against the Rfam (http://rfam.sanger.ac.uk/) to eliminate non-coding RNA tags (rRNA, tRNA, snRNA, snoRNA), repeat sequences, and protein-coding genes. And the remaining clean reads were mapped to miRNA sequences in miRBase v. 22.0 [43] to identify known miRNAs. Unmapped sequences in the miRbase database were aligned to the *C. camphora* genome [31] to identify novel candidate miRNAs by using MiREvo and miRDeep2 software [44, 45]. To confirm putative miRNAs in *C. camphora*, the hairpin RNA structures containing sequences were predicated from the flank 120nt sequences using RNAfold software (http://rna.tbi.univie.ac.at/cgi-bin/RNAWebSuite/RNAfold.cgi). The criteria for secondary structure prediction were: (1) number of nucleotides in one bulge in stem (≤ 12), (2) number of base pairs in the stem region of the predicted hairpin (≥ 16), (3) cutoff of free energy (kCal/mol ≤−15), (4) length of hairpin (up and down stems + terminal loop ≥ 50), (5) length of hairpin loop (≤ 200), (6) number of nucleotides in one bulge in mature region (≤ 4), (7) number of biased errors in one bulge in mature region (≤ 2), (8) number of biased bulges in mature region (≤ 2), (9) number of errors in mature region (≤ 4), (10) number of base pairs in the mature region of the predicted hairpin (≥ 12), (11) percent of mature in stem (≥ 80). Noncoding sequences capable of forming a stem-loop structure and meeting the established miRNA prediction criteria [46]were classified as the true novel miRNAs of *C. camphora*. The miRNA expression level was quantified using the transcripts per million (TPM) method. Differentially expressed miRNAs (DEMs) between any two groups was conducted using the DESeq2 R package (version 1.16.1). miRNAs with *p*-value of < 0.05 and an absolute log2(fold change) value greater than 1 were considered statistically significant.

### RT-qPCR analysis of miRNAs

We conducted to verify the expression levels of nine miRNAs in H_MAR_1, 2, 3, H_MAY_1, 2, 3, and L_MAY_1, 2, 3 by real-time quantitative PCR (RT-qPCR). Reverse transcription was performed using the Hifair miRNA 1 st Strand cDNA Synthesis Kit (YEASEN) following the manufacturer’s instructions. RT-qPCR reactions were performed using the Hieff UNICON Universal Blue qPCR SYBR Green Master Mix (YEASEN) in triplicate. Cycle threshold (Ct) values were recorded using the CFX96 platform (Bio-Rad, China) in accordance with the provided protocol. Nine mature miRNA sequences were used as forward primers (Table S1) and synthesized by Sangon Biotech (Shanghai) Co., Ltd., China. Universal reverse primers and the reference gene (U6) were supplied by the Hifair miRNA 1 st Strand cDNA Synthesis Kit (YEASEN). miRNA expression levels were normalized using the 2^−ΔΔCt^ method.

### Dual-luciferase reporter assay

The wild-type and synonymous mutant sequences of the MicroRNA Response Element (MRE) for *Cca.gene38630* (*SPL6*) and *Cca.gene34720* (*GGDR*)were cloned into the pmriGLO vector, resulting in the recombinant plasmids pmriGLO:*SPL6*, pmriGLO:*SPL6*-*mt*, pmriGLO:*GGDR*, and pmriGLO:*GGDR*-*mt*. Concurrently, overexpression vectors for ppe-miR156f_L + 1 and gma-miR5368-p5_1ss1TC were constructed, yielding the recombinant plasmids 35 S::miR156f and 35 S::miR5368. Following the manufacturer’s instructions of the Vazyme Dual Luciferase Reporter Assay Kit, Arabidopsis thaliana protoplasts were transfected with the constructed plasmids. The LUC fluorescence signals were measured from three replicate wells per sample using a GLOMax 96 microplate luminometer. The average firefly luciferase (FLUC) and Renilla luciferase (RLUC) luminescence values were calculated, and the FLUC intensity was normalized to RLUC (expressed as the FLUC/RLUC ratio).

### Target gene identification by degradome sequencing

Poly(A) RNA was purified from nine RNA samples (20 µg) by using poly-T oligo-attached magnetic beads using two rounds of purification to construct the degradome sequencing library. Because the 3’ cleavage product of the mRNA contains a 5’ monophosphate, the 5’ adapters were ligated to the 5’ end of the 3’ cleavage product of the mRNA by the RNA ligase. The next step is reverse transcription to make the first strand of cDNA with a 3’ adapter random primer, and size selection was performed with AMPureXP beads. Then the cDNAs were amplified with PCR. The average insert size for the final cDNA library was 200–400 bp. Single-end sequencing (50 bp) was performed on Illumina HiSeq 2500 following the protocol recommended by Lianchuan Biotechnology Co., Ltd., China. Raw data were filtered using Illumina Pipeline v1.5 software. miRNA target genes and their cleavage sites were predicted using CleaveLand v. 4.3 [47]. Oligomap was used to map the degradome data to the reference transcriptome and construct a degradome density file. The standard sequences of value for the degradome were compared in the NRPM database (per million reads) to remove redundancy. Target genes paired with miRNA sequences were predicted by GSTAr. Finally, the results of the two software programs were integrated to determine the common mRNA that was the target of the miRNA. The T-plot of the miRNA-target pairs was plotted based on degradome density files. On the basis of read abundance at cleavage sites (RCSs), miRNA targets were categorized into five groups (0 to 4), reflecting decreasing reliability.

### Enrichment analysis of targets

To investigate the biological significance of miRNAs, the target genes of DEMs were analyzed for enrichment in the Gene Ontology (GO) and Kyoto Encyclopedia of Genes and Genomes (KEGG) pathways. GO and KEGG pathway enrichment analyses were performed using the GOseq R package and KOBAS 2.0 software [48], respectively. *P*-value < 0.05 were used to test the statistical enrichment of GO terms and KEGG pathways.

## Supplementary Information

Additional File 1: Figure S1 Conservation Profile of the identified miRNAs. Additional file 2: Figure S2 miRNA expression calorimetry map. The expression profiles of all miRNAs in H_MAR and H_MAY. Abundance is demonstrated with a color gradient by normalized log2-transformed values. Blue indicates low expression, and red indicates high expression. Additional file 3: Figure S3 miRNA expression calorimetry map. The expression profiles of all miRNAs in H_MAY and L_MAY. Abundance is demonstrated with a color gradient by normalized log2-transformed values. Blue indicates low expression, and red indicates high expression. Additional file 4: Figure S4 The regulatory network of DEMs and their target genes. Pink nodes indicate miRNAs and yellow nodes indicate target genes. Additional file 5: Figure S5 Analysis of TPM values for selected miRNAs and their target Genes.

Additional File 2: Table S1. RT-qPCR primers of miRNAs. Additional file 7: Table S2. sRNA deep sequencing profiles of the nine simples of *C.camphora*. Additional file 8: Table S3. Sequence of mature and pre-miRNA. Additional file 9: Table S4. Predicted miRNAs and hairpains in each sample. Additional file 10: Table S5. Conservation profile of the identified miRNAs. Additional file 11: Table S6. Conservation of the identified miRNA with other species. Additional file 12: Table S7. The expression profile of miRNA. Additional file 13: Table S8. Differentially expressed miRNAs in the H_MAR, H_MAY, and L_MAY. Additional file 14: Table S9. Target genes of miRNA Identification and Expression.

## Data Availability

The datasets generated or analysed during the current study are available in the supplementary materials and Genome Sequence Archive repository, GSA: CRA025779.
