# Supplementary figures and images for: Integrative analysis of small RNA and degradome sequencing reveals the role of miRNAs in monoterpene biosynthesis in linalool-type Cinnamomum camphora
Source: BMC Plant Biol. 2025 Nov 11;25:1536. doi: 10.1186/s12870-025-07588-2 (PMC12606813; doi:10.1186/s12870-025-07588-2)

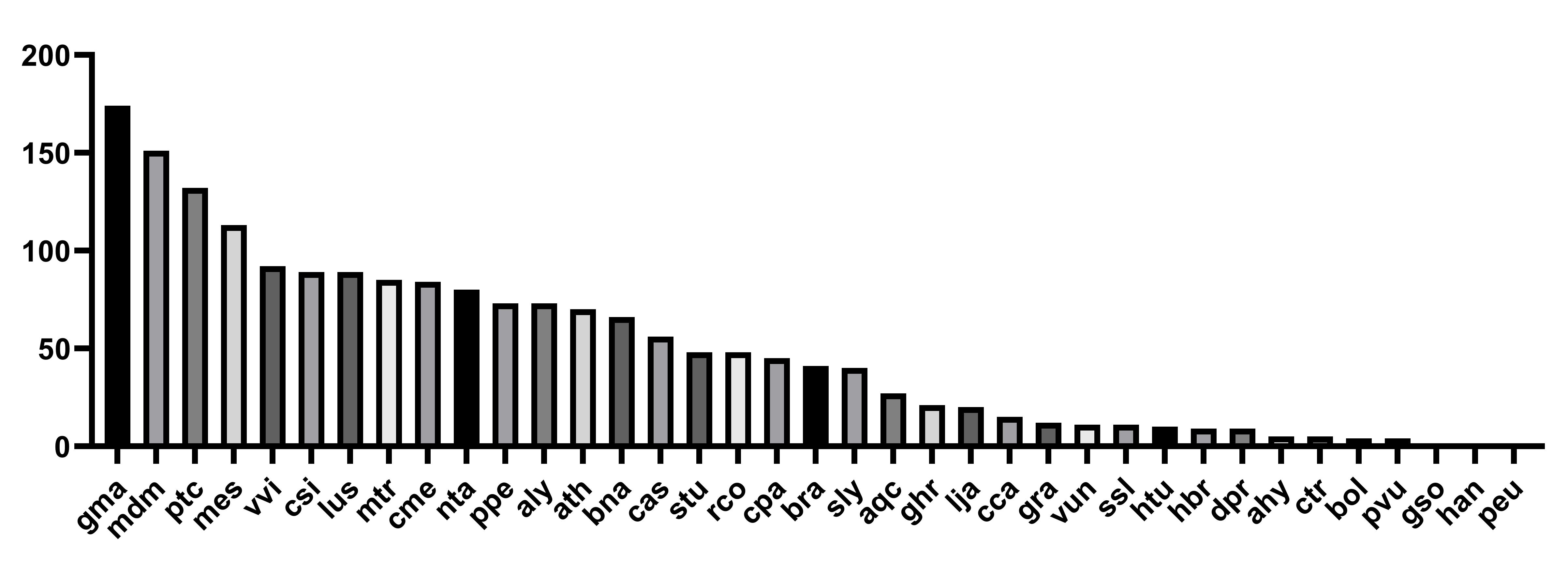

Supplement: Supplementary file 1 — Additional File 1: Figure S1 Conservation Profile of the identified miRNAs. Additional file 2: Figure S2 miRNA expression calorimetry map. The expression profiles of all miRNAs in H_MAR and H_MAY. Abundance is demonstrated with a color gradient by normalized log2-transformed values. Blue indicates low expression, and red indicates high expression. Additional file 3: Figure S3 miRNA expression calorimetry map. The expression profiles of all miRNAs in H_MAY and L_MAY. Abundance is demonstrated with a color gradient by normalized log2-transformed values. Blue indicates low expression, and red indicates high expression. Additional file 4: Figure S4 The regulatory network of DEMs and their target genes. Pink nodes indicate miRNAs and yellow nodes indicate target genes. Additional file 5: Figure S5 Analysis of TPM values for selected miRNAs and their target Genes. [file 12870_2025_7588_MOESM1_ESM.zip › Figure S1.jpg]

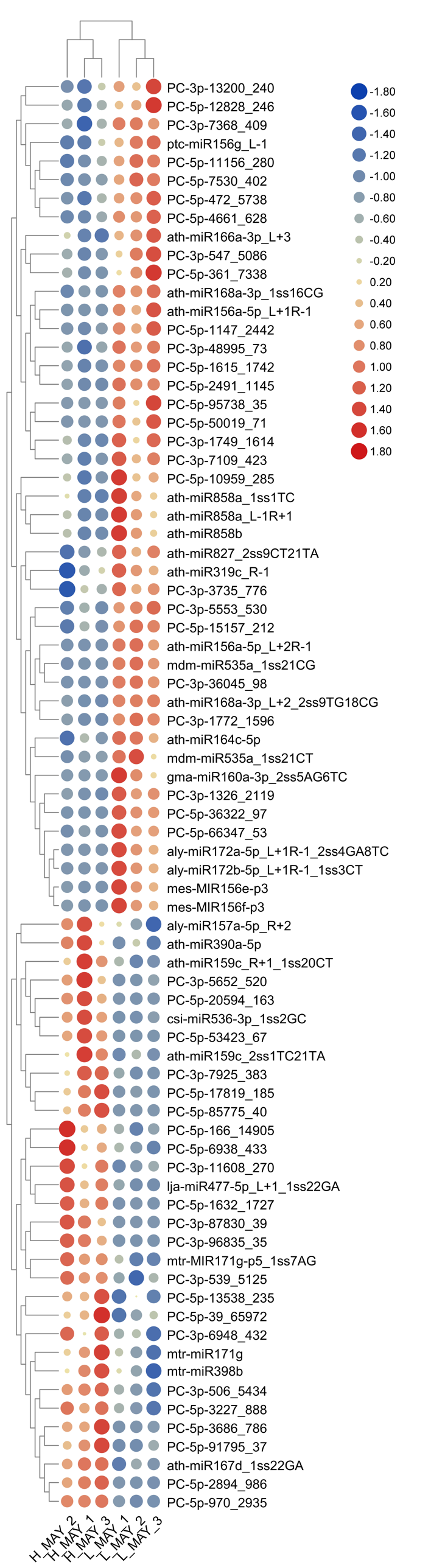

Supplement: Supplementary file 1 — Additional File 1: Figure S1 Conservation Profile of the identified miRNAs. Additional file 2: Figure S2 miRNA expression calorimetry map. The expression profiles of all miRNAs in H_MAR and H_MAY. Abundance is demonstrated with a color gradient by normalized log2-transformed values. Blue indicates low expression, and red indicates high expression. Additional file 3: Figure S3 miRNA expression calorimetry map. The expression profiles of all miRNAs in H_MAY and L_MAY. Abundance is demonstrated with a color gradient by normalized log2-transformed values. Blue indicates low expression, and red indicates high expression. Additional file 4: Figure S4 The regulatory network of DEMs and their target genes. Pink nodes indicate miRNAs and yellow nodes indicate target genes. Additional file 5: Figure S5 Analysis of TPM values for selected miRNAs and their target Genes. [file 12870_2025_7588_MOESM1_ESM.zip › Figure S3.jpg]

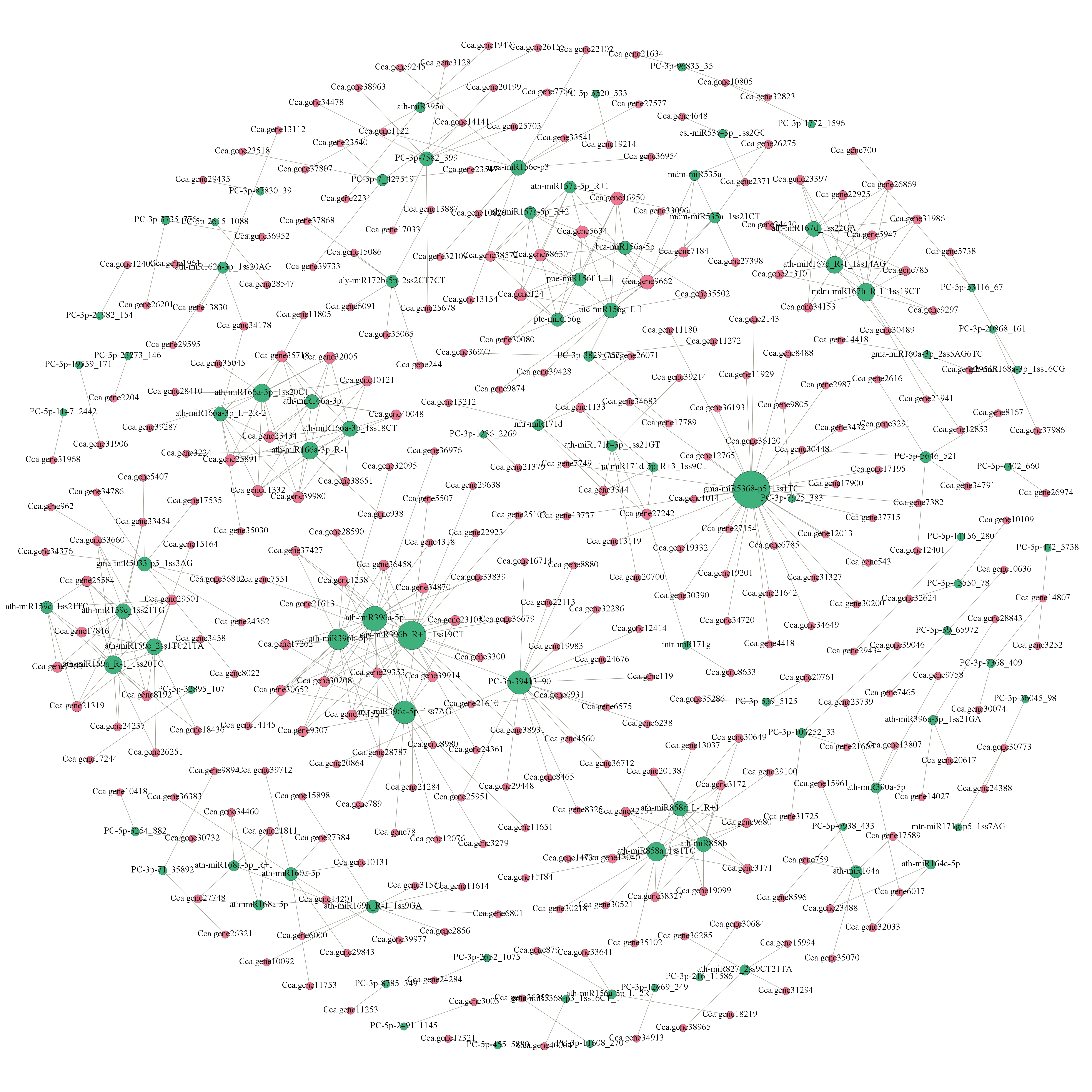

Supplement: Supplementary file 1 — Additional File 1: Figure S1 Conservation Profile of the identified miRNAs. Additional file 2: Figure S2 miRNA expression calorimetry map. The expression profiles of all miRNAs in H_MAR and H_MAY. Abundance is demonstrated with a color gradient by normalized log2-transformed values. Blue indicates low expression, and red indicates high expression. Additional file 3: Figure S3 miRNA expression calorimetry map. The expression profiles of all miRNAs in H_MAY and L_MAY. Abundance is demonstrated with a color gradient by normalized log2-transformed values. Blue indicates low expression, and red indicates high expression. Additional file 4: Figure S4 The regulatory network of DEMs and their target genes. Pink nodes indicate miRNAs and yellow nodes indicate target genes. Additional file 5: Figure S5 Analysis of TPM values for selected miRNAs and their target Genes. [file 12870_2025_7588_MOESM1_ESM.zip › Figure S4.jpg]

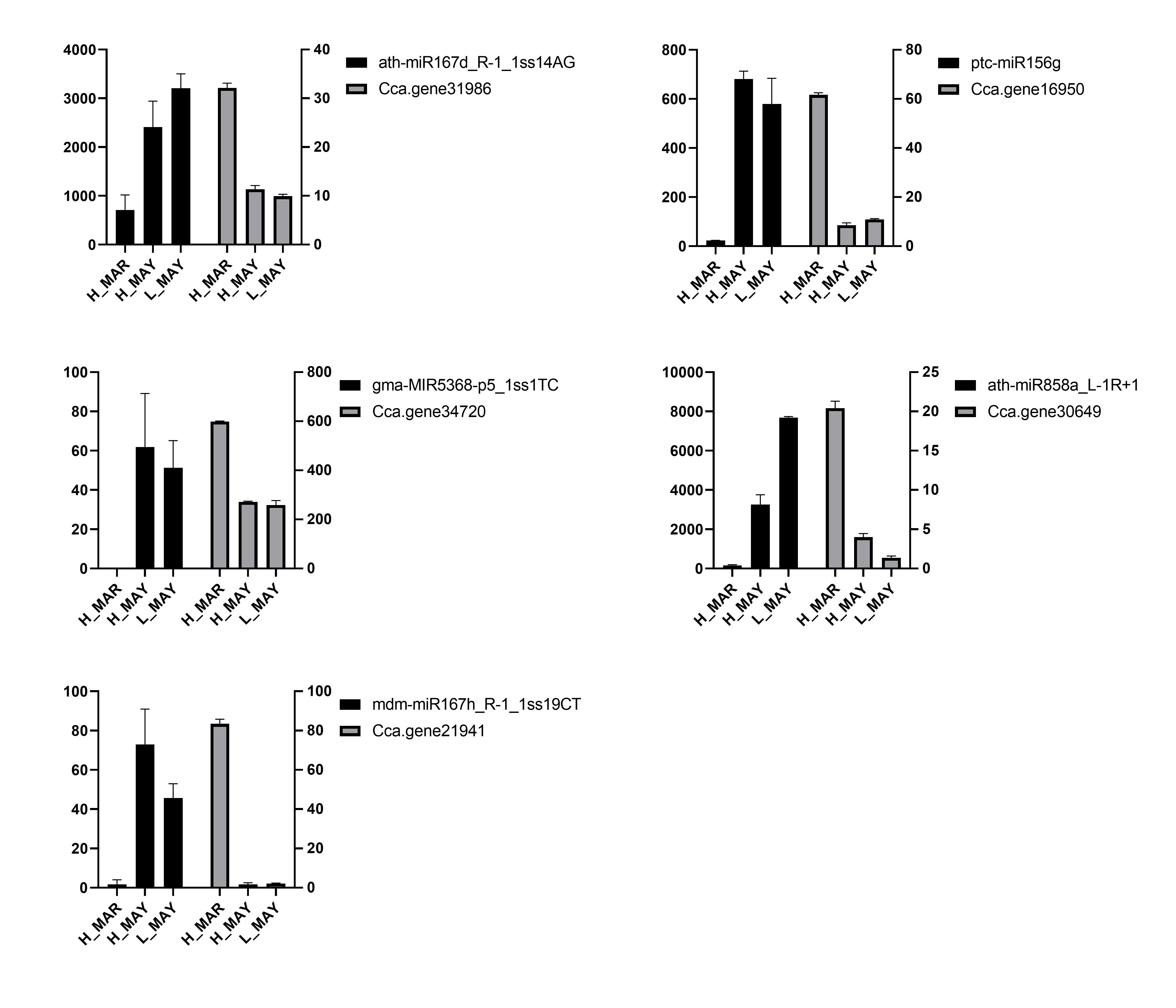

Supplement: Supplementary file 1 — Additional File 1: Figure S1 Conservation Profile of the identified miRNAs. Additional file 2: Figure S2 miRNA expression calorimetry map. The expression profiles of all miRNAs in H_MAR and H_MAY. Abundance is demonstrated with a color gradient by normalized log2-transformed values. Blue indicates low expression, and red indicates high expression. Additional file 3: Figure S3 miRNA expression calorimetry map. The expression profiles of all miRNAs in H_MAY and L_MAY. Abundance is demonstrated with a color gradient by normalized log2-transformed values. Blue indicates low expression, and red indicates high expression. Additional file 4: Figure S4 The regulatory network of DEMs and their target genes. Pink nodes indicate miRNAs and yellow nodes indicate target genes. Additional file 5: Figure S5 Analysis of TPM values for selected miRNAs and their target Genes. [file 12870_2025_7588_MOESM1_ESM.zip › Figure S5.jpg]
